# 

**Published:** 1847-01

**Authors:** 


### CONTENTS OF N° XLV

OF THE

### British and Foreign Medical Review.

JANUARY, 1847.

34546

### PART FIRST.—Analytical and Critical Reviews.

PAGE

ART. I.—1. An Experimental and Critical Inquiry into the Nature and Treatment of Wounds of the Intestines; illustrated by Engravings. By SAMUEL D. GROSS, M.D., Professor of Surgery in the Louisville Medical Institute, Surgeon to the Louisville Marine Hospital, &c. . . . . . 1

2. Recherches sur l'Emploi d'un Nouveau Procédé de Suture contre les Divisions de l'Intestin et sur la possibilité de l'Adossement de cet Organe avec lui-même dans certaines Blessures. Par J. A. GELY (de Nantes), M.D. Mémoire adressé à l'Académie Royale de Médecine . . . ib.

Researches on the Application of a New Method of Suture in Wounds of the Intestines, and on the possibility of inflecting the Intestine on itself in certain Wounds of that Organ. By J. A. GELY, M.D., of Nantes. Memoir presented to the Royal Academy of Medicine of Paris.

3. Die Operative Chirurgie. Von JOHANN F. DIEFFENBACH. Erster Band. Capitel LXII. Operation des widernaturlichen Afters . . ib.

ART. II.—Nosologia Positiva. Scritta da VINCENZIO LANZA, Dottore in Medicina, Professore nella Cattedra di Medicina Pratica della Regia Universita' degli Studii di Napoli, Medico Primario e Direttore della Clinica dello Spedale della Pace, &c. &c. . . . . . . . . 37

Positive Nosology. By V. LANZA, M.D., Professor of the Practice of Physic in the Royal University of Naples, &c.

ART. III.—Elements of Pathological Anatomy. By SAMUEL D. GROSS, M.D., Professor of Pathological Anatomy in the Medical Department of the Cincinnati College, &c. Second edition . . . . . . 63

ART. IV.—Recueil de Mémoires de Médecine, de Chirurgie, et de Pharmacie Militaires; redigé sous la Surveillance du Conseil de Santé des Armées. Publié par Ordre de S. Ex. le Ministre Secrétaire de l'Etat au Département de la Guerre . . . . . . . . 73

Collection of Memoirs on Military Medicine, Surgery, and Pharmacy. Published officially. Fifty-eight Volumes.

ART. V.—1. Histoire de la Médecine depuis son Origine jusqu'au XIXe Siècle. Par le Docteur P. V. RENOUARD. Tom. ii . . . . . 85

2. Saggio sopra il Modo e le Regole di osservare e massime in Medicina. Del Dr. L. EMILIANI, Prof. Emer. di Clinica Medica e Medicina Pratica nella Regia Universita di Modena, &c. . . . . ib.

3. Zur Charakteristik der Medicin der Gegenwart. Von Dr. J. M. LEUPOLDT, offentl. ord. Professor an der Universität zu Erlangen . . ib.

4. The Medical Police of the United Kingdom. From the Westminster Review for March . . . . . . . ib.

§

PAGE

ART. VI.—Clinical Illustrations of the Diseases of India, as exhibited in the Medical History of a Body of European Soldiers for a series of years from their arrival in that Country. By WILLIAM GEDDES, M.D., &c. &c., late Surgeon of the Madras European Regiment . . . . . 106

ART. VII.—1. Der Speichel in physiologischer, diagnostischer und therapeutischer Beziehung. Eine mit Anmerkungen vermehrte Bearbeitung, nach S. WRIGHT, M.D., &c. . . . . . . . . . 129

2. Het Speeksel uit een physiologisch, diagnostisch, en therapeutisch oogpunt beschouwd, döor S. WRIGHT, M.D. Naar de Hoogduitsche bearbeiding van Dr. S. ECKSTEIN, vertaild en met aanteekeningen voorzien door F. REINDERHOFF . . . . . . . . ib.

ART. VIII.—Anatomie de Texture ou Histologie, appliquée à la Physiologie et à la Pathologie. Par AD. BURGGRAEVE, Professeur à la Faculté de Médecine de l'Université de Gand, &c. . . . . . . 136

Textural Anatomy, or Histology applied to Physiology and Pathology. By AD. BURGGRAEVE, Professor at Gand.

ART. IX.—Remarks on the Dysentery and Hepatitis of India. By E. A. PARKES, M.B., late Assistant Surgeon H. M. 84th Regiment . . . 146

ART. X.—Malgaigne's Operative Surgery. Translated from the French, by F. BRITTAN, A.B., M.D., &c. &c. . . . . . 155

ART. XI.—1. Recherches Expérimentales sur le Développement de la Graisse pendant l'Alimentation des Animaux. Par M. BOUSSINGAULT. (Annales de Chimie, tom. xiv, 1845) . . . . . 157

2. Der Chemische Process der Respiration. Von JUSTUS VON LIEBIG. (Annalen der Chemie, Band lviii, 1846) . . . . . ib.

3. Experimental Researches on the Food of Animals and Fattening of Cattle, with remarks on the Food of Man. By ROBERT DUNDAS THOMSON, M.D. . ib.

ART. XII.—The Military Miscellany; comprehending a History of the Recruiting of the Army, Military Punishments, &c. &c. By HENRY MARSHALL, F.R.S.E., Deputy Inspector-General of Army Hospitals, &c. &c. . . 163

ART. XIII.—Lectures on Subjects connected with Clinical Medicine, comprising Diseases of the Heart. By P. M. LATHAM, M.D., &c. Vol. II . 169

ART. XIV.—Practical Observations and Suggestions in Medicine. By MARSHALL HALL, M.D., F.R.S., L. & E., &c. &c. First and second Series . . 185

ART. XV.—Du Hachisch et de l'Aliénation Mentale, Etudes Psychologiques. Par J. MOREAU (de Tours), Médecin de l'Hospice de Bicêtre, Membre de la Société Orientale de Paris . , . . . 217

Psychological Studies on Hachisch and on Mental Derangement. By J. MOREAU (of Tours), Physician to the Hospital of Bicêtre, and Member of the Oriental Society of Paris.

### PART SECOND.—Bibliographical Notices.

ART. I.—Dr. Underwood's Treatise on the Diseases of Children; with Directions for the Management of Infants. Tenth Edition, with Additions. By HENRY DAVIES, M.D., Senior Physician to the British Lying-in Hospital . 237

ART. II.—Chemistry of the Four Seasons. By THOMAS GRIFFITHS . . 238

ART. III.—A Practical Treatise on the Diseases Peculiar to Women. By SAMUEL ASHWELL, M.D., &c. Second Edition . . . . . 239

ART. IV.—The Health of Towns as influenced by defective Cleansing and Drainage; and on the application of the Refuse of Towns to Agricultural Purposes; being a Lecture delivered at the Russell Institution, May 5, 1846. By WILLIAM A. GUY, M.B., Cantab, &c. &c. . . . . . 240

PAGE

ART. V.—Fever physiologically considered; considerations on Yellow Fever, Typhus Fever, Plague, Cholera, and Sea-Scurvy; also the Questions of Contagion and the Quarantine Laws; with an Address to the Public, &c., on the popular Treatment of Cholera. By DAVID M'CONNELL REED, Esq., Licentiate of Medicine, &c. . . . . . . . 241

ART. VI.—1. Introductory Discourse on the Mode of Investigating the Sciences belonging to the Medical Profession, delivered in the Theatre of St. George's Hospital, October 1, 1846. By Sir BENJAMIN C. BRODIE, Bart. &c. . 242

2. On Medical Education; being a Lecture delivered at King's College, London, at the opening of the Medical Session, 1846-7, to which is added a Lecture delivered on the same occasion in the year 1842. By WILLIAM A. GUY, M.B. Cantab., Professor of Forensic Medicine, King's College, &c. . . ib.

3. The Motives of Industry in the Study of Medicine; an Address delivered at St. Bartholomew's Hospital, on Thursday, October 1, 1846. By JAMES PAGET, F.R.C.S., Warden of the College, and Lecturer on Physiology in the Hospital . . . . . . . . ib.

ART. VII.—A Manual of Materia Medica and Therapeutics, including the Preparations of the Pharmacopœias of London, Edinburgh, and Dublin, with many new Medicines. By J. FORBES ROYLE, M.D., F.R.S., Professor of Materia Medica and Therapeutics, King's College, London . . 244

ART. VIII.—Principles of Human Physiology, with their chief applications to Pathology, Hygiene, and Forensic Medicine. By W. B. CARPENTER, M.D., F.R.S., Fullerian Professor of Physiology to the Royal Institution of Great Britain. Third Edition . . . . . . . 245

ART. IX.—A Manual of the Principles and Practice of Ophthalmic Medicine and Surgery. By T. WHARTON JONES, F.R.S., Lecturer on Anatomy. Physiology, and Pathology, at the Charing-cross Hospital, &c. . . . 246

ART. X.—On the Correlation of Physical Forces: being the substance of a Course of Lectures delivered in the London Institution, in the year 1843. By R. W. GROVE, M.A., F.R.S, Barrister-at-Law . . . . 247

ART. XI.—Urinary Deposits; their Diagnosis, Pathology, and Therapeutical Indications. By GOLDING BIRD, A.M., M.D., F.R.S., &c. Second Edition . 248

ART. XII.—A descriptive Catalogue of the Anatomical Museum of St. Bartholomew's Hospital. Vol. I, containing the description of the specimens illustrative of Pathological Anatomy . , . . 249

ART. XIII.—Records of Harvey: in Extracts from the Journals of the Royal Hospital of St. Bartholomew. With Notes. By JAMES PAGET, Warden of the Collegiate Establishment, and Lecturer on Physiology in the Hospital . ib.

ART. XIV.—Lectures and Observations on Clinical Surgery. By ANDREW ELLIS, F.R.C.S.I., &c. . . . . . . 250

ART. XV.—The Pathological Anatomy of the Human Body. By JULIUS VOGEL, M.D. Translated by G. E. DAY, M.A. and L.M. Cantab. . . ib.

ART. XVI.—Works published by the Sydenham Society:—

1 An Anatomical Description of the Diseases of the Organs of Respiration. By C. E. HASSE, M.D. Translated and edited by W. E. SWAINE, M.D. . 251

2. The Seven Books of Paulus Ægineta. Translated from the Greek, with a Commentary. By FRANCIS ADAMS. Vol. II . . . ib.

3. The Works of William Hewson, F.R.S. Edited with an Introduction and Notes, by GEORGE GULLIVER, F.R.S. . . . . ib.

ART. XVII.—On Diseases of the Skin. By ERASMUS WILSON, F.R.S. Second ed. ib.

ART. XVIII.—Knowledge; an Introductory Address, delivered at the Bristol Athenæum, October 5, 1846, by JOHN ADDINGTON SYMONDS, M.D. . 252

ART. XIX.—The Microscopic Anatomy of the Human Body in Health and Disease. By ARTHUR HILL HASSALL, F.L.S., M.R.C.S, &c. Parts III-V . ib.

PAGE

Art. XX.—Œuvres Complètes d'Hippocrate, Traduction Nouvelle, avec le Texte Grec en regard, collationé sur les Manuscripts et toutes les Editions, &c. Par E. Littré . . . . . . 253
The Complete Works of Hippocrates, &c. By E. Littré, &c.

Art. XXI.—Remarks on National Education. By George Combe . . 254

Art. XXII.—A Practical Manual, containing a Description of the General, Chemical, and Microscopical Characters of the Blood and Secretions of the Human Body, as well as of their Components, including both their Healthy and Diseased States, with the best methods of separating and estimating their Ingredients; also, a succinct Account of the various Concretions occasionally found in the Body, and forming Calculi. By John W. Griffiths, m.d., f.l.s., Senior Physician to the Finsbury Dispensary. Part II . . ib.

Art. XXIII.—Handbook of Human Anatomy, General, Special, and Topographical. Translated from the original German of Dr. Alfred von Behr, and adapted to the use of the English Student. By John Birkett, Fellow of the Royal College of Surgeons of England, and Demonstrator of Anatomy at Guy's Hospital . . . . . . . 255

Art. XXIV.—An Essay on the Treatment of Compound and Complicated Fractures, being an Annual Address before the Massachusetts Medical Society, in Boston, U. S. America, May 28, 1845. By William J. Walker, m.d. 256

### Part Third.—Natural History and Treatment of Diseases.

I.—On the Observation of Nature in the Study and Treatment of Disease. By Andrew Combe, m.d. In a Letter to John Forbes, m.d., f.r.s. . 257

II.—Notes on some Experiments, illustrating the Influence of the Vis Medicatrix, and of the Imagination, in the Cure of Diseases. By a Naval Surgeon. In a Letter to John Forbes, m.d., f.r.s. . . . . 265

III.—On the Treatment of Fever by the Application of Cold Water to the Surface of the Body. By J. H. Stallard, Esq., Surgeon to the Leicester General Dispensary. In a Letter to John Forbes, m.d., f.r.s. . . 269

### Part Fourth.—Original Reports and Memoirs.

Report on the Progress of Practical Medicine, in the departments of Midwifery and the Diseases of Women and Children, in the years 1845-6. By Charles West, m.d., Member of the Royal College of Physicians, Physician-Accoucheur and Lecturer on Midwifery to the Middlesex Hospital, and Senior Physician to the Royal Infirmary for Children . . . 277

On a New Means of rendering Surgical Operations Painless . . . 309

